# Deployment Protection for Interference of 5G Base Stations with Aeronautical Radio Altimeters

**DOI:** 10.3390/s24072313

**Published:** 2024-04-05

**Authors:** Zhaobin Duan, Zhenyang Ma, Jie Bai, Peng Wang, Ke Xu, Shun Yuan

**Affiliations:** 1 Key Lab of Civil Aircraft Airworthiness Technology, Tianjin 300300, China; zbduan@cauc.edu.cn (Z.D.);; 2 Institute of Technology and Innovation, Civil Aviation University of China, Tianjin 300300, China; 3 College of Safety Science and Engineering, Civil Aviation University of China, Tianjin 300300, China

**Keywords:** radio altimeter, 5G, interference, deployment protection

## Abstract

In this manuscript, we present a novel deployment protection method aimed at safeguarding aeronautical radio altimeters (RAs) from interference caused by fifth-generation (5G) telecommunication base stations (BSs). Our methodology involves an integrated interference model for defining prohibited zones and utilizes power control and angle shutoff methods to mitigate interference. First, to ensure reliable protection, we define both horizontal and vertical prohibited zones and investigate their variations to immunize RA against 5G interference. Second, we validate the effectiveness of the model in various operational scenarios, analyzing the influence of factors such as base station types, antenna parameters, flight altitude, and aircraft attitudes to cover a wide range of real-world scenarios. Third, to mitigate interference, we propose and analyze the power control and angle shutoff methods through simulation for the RMa prohibited zone. Our results demonstrate the efficacy of the deployment protection method in safeguarding RAs from 5G interference, providing guidance for interference protection during civil aviation operations and base station deployment near airports.

## 1. Introduction

The interference of 5G base stations with aeronautical radio altimeters is a hot topic in the aviation industry. Experimental evidence from relevant research institutions has thoroughly validated the existence of this interference effect at the center frequency of 3.93 GHz [1,2,3]. Radio altimeters are critical aviation safety systems that are mandatory equipment on aircraft. A radio altimeter is the only system on an aircraft used to determine the altitude above ground level (AGL) of the aircraft. The altitude information provided by the radio altimeter is fundamental to a series of safety-related aircraft operations and navigation functions, including the TAWS (Terrain Awareness Warning System), TCAS (Traffic Collision Avoidance System), wind shear detection, flight control, autoland systems, and precision approaches. Any harmful interference with the functionality of the radio altimeter during any phase of flight can pose serious safety risks to passengers, crew, and ground personnel [3].

**Figure 1:**
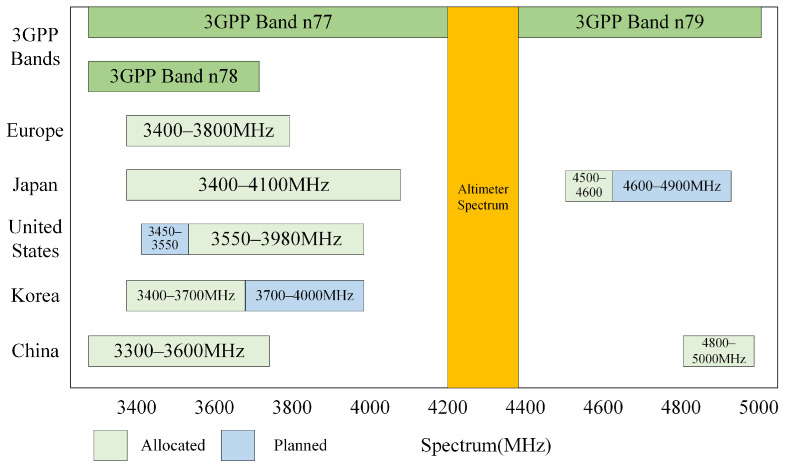
Spectrum allocation of 5G and RAs.

**Figure 2:**
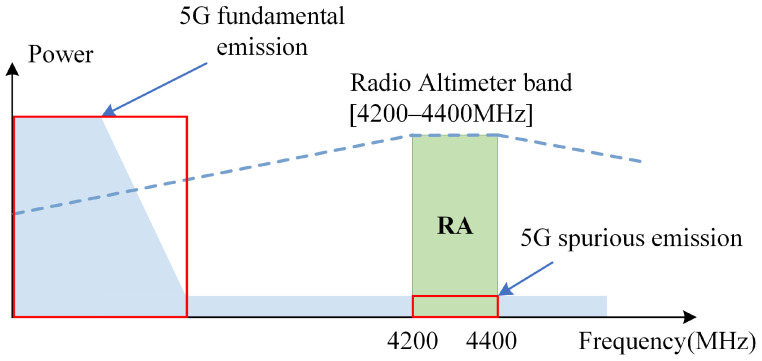
Interference mechanism.

However, with the emergence of numerous new 5G telecommunication systems in the vicinity of the operating frequency of radio altimeters (Figure 1), there is a risk of both out-of-band and in-band interference with radio altimeters caused by the fundamental emissions and spurious emissions of 5G [1] (Figure 2). The development and initial application of radio altimeters took place in the 1970s. At that time, there were fewer interference systems operating in the frequency range of RAs. As a result, the international standards for radio altimeters, including RTCA DO-155 [4], EUROCAE ED-30 [5], and ARINC707-7 [6], did not impose constraints in terms of the resistance of RAs to interference. These standards also did not address the deployment of protection against 5G interference [7,8,9].

As a result, the Federal Aviation Administration (FAA) released a grand total of eighteen statements regarding the 5G network between 7 December 2021 and 23 February 2022. Moreover, on 25 March 2021, the International Civil Aviation Organization (ICAO) disseminated a letter of concern to all member states regarding the potential for interference with aircraft radio altimeters. International Telecommunication Union (ITU) Report 2059-0 scrutinizes the interference mechanism of 5G with RAs with meticulous attention to detail [1]. Furthermore, the AVSI (Airborne Vehicle Systems Institute) has specifically published reports that determine interference threshold values through experimental methods [2].

**Figure 3:**
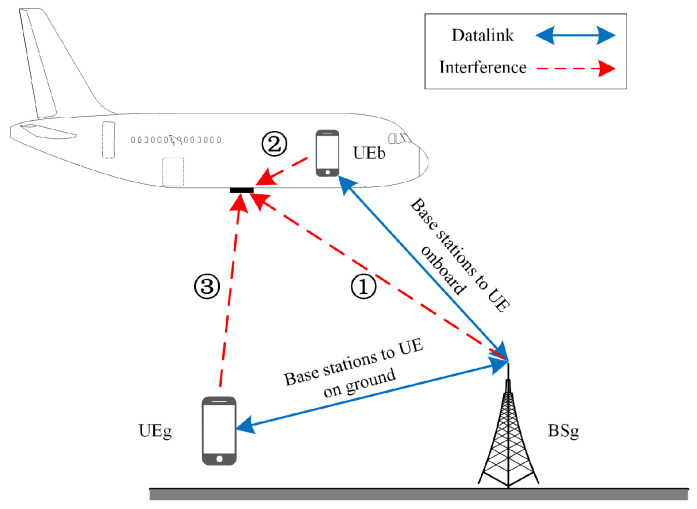
Interference scenarios.

The issue of 5G interference with RAs can be classified into the following three main scenarios: ① interference from 5G base stations (BSg), ② interference from 5G user equipment on board (UEb), and ③ interference from 5G user equipment on the ground (UEg), as shown in Figure 3 [10,11]. In this study, we primarily focus on the interference of 5G base stations with radio altimeters and the fundamental 5G emission.

The impact of 5G interference on radio altimeters is a novel and critical problem. It poses a significant challenge to deploy base stations surrounding airports. There are existing studies on 5G interference in the field of communication [12]. However, these studies mainly focused on specific application scenarios, such as urban base station deployment and satellite communication interference. Ref. [13] investigated covert mmWave communications with a finite blocklength, studying both phase array and linear frequency diverse array beamforming schemes to enhance antenna gain. Ref. [14] proposed an energy-efficient and secure transmission design for integrated terrestrial–aerial networks (ITAN) using a multi-layer reconfigurable intelligent surface (RIS). Ref. [15] carried out sharing studies between advanced international mobile telecommunication systems and geostationary satellite networks. Refs. [16,17] demonstrated that distance protection schemes could mitigate 5G interference with FSS (Fixed-Satellite Service) effectively. Ref. [18] examined the transmitting power of 5G base stations and its impact on fixed-satellite services, utilizing a C-band receiver antenna. Ref. [19] employed unmanned aerial vehicles (UAVs) to measure the interference caused by 5G base stations, presenting a more comprehensive interference picture than conventional methods. Ref. [20] used Monte Carlo methods and minimum coupling loss to calculate the protection distance of 5G networks from FSS. Ref. [21] focused on the issue of 5G interference in future Non-Terrestrial Networks (NTNs). They proposed two mathematical frameworks to address the problems of inter-beam and inter-satellite interference in satellite 5G beam systems. Refs. [22,23] conducted a study on the interference generated by a Wireless Avionics Intra-Communications (WAIC) system utilizing 5G technology. However, these studies have not fully resolved the issue of interference with radio altimeters in aviation. The main problems are described as follows.

1. They have not yet proposed an integrated interference model tailored to the complex operational scenarios of aircraft, nor have they provided a method for calculating protective measures in accordance with aviation safety requirements.
2. They have not adequately explored the variations of protection zones under dynamic aircraft operational scenarios, nor have they proposed deployment protection measures for dynamic situations. Moreover, their discussion of path loss is overly simplistic.
3. They have failed to provide an effective and direct analytical framework for addressing the deployment of 5G base stations near airports and to offer direct mitigation measures for base station deployment.

To address these challenges, we approach the problem from the perspective of aviation operational safety. Direct recommendations can be provided for the establishment of prohibited zones and the implementation of interference mitigation measures for base station deployments near airports. The main differences between this study and previous work are summarized in Table 1. The primary contributions of this work are described as follows.

1. First, we propose a novel deployment protection method to define prohibited zones, considering aviation safety requirements such as the WCLS (Worst-Case Landing Scenario) and ICAO 6 dB margin, and conduct analysis for both horizontal and vertical prohibited zones. The adoption of an integrated computational model addresses issues where other analytical models are not applicable to civil aviation operations.
2. Second, we investigate the variations of prohibited zones during aircraft dynamic operations, considering factors such as an aircraft’s roll/pitch angle, altitude AGL, and the stochastic feature of path loss. We examine three different base stations (rural macrocells (RMas), urban macrocells (UMas), and urban microcells (UMis)), two antenna configurations (an omnidirectional antenna and 16 × 16 beamforming antenna arrays), and the deployment height and vertical scan angle of the base station.
3. Finally, we discuss methods for mitigating interference within the prohibited zones using beamforming technology, employing power control and angle shutoff techniques to protect radio altimeters. This provides a new approach for deploying 5G base stations near airports while ensuring aviation safety.

**Table 1:** The differences between this paper and previous works.

		This Paper	[3]	[17]	[18]	[20]	[24]	[25]
Integrated Interference Model	Applicable in aviation	◯	◯					
	RMa, UMa, UMi, ICAO 6 dB, WCLS, antenna types	◯			◯			◯
	Prohibited zones	◯		◯		◯		
Dynamic Aircraft Operational Scenarios	Aircraft altitude and attitude	◯						
	Dynamic interference thresholds	◯	◯			◯	◯	
	Vertical prohibited zones	◯						
Path Loss	Analyzing LOS and NLOS scenarios	◯		◯	◯			
Mitigation Measures	Applicable for BSs near airports	◯	◯	◯			◯	◯
	Tx power control and angle shutoff	◯						◯

## 2. Methodology

### 2.1. Integrated Interference Model

In the practical scenarios of aviation operations, deploying protection requires an integrated modeling approach for all factors. The interference power between an interfering transmitter (*Tx*) and a victim receiver (*Rx*) is described as Equation (1) [25,26,27].

(1) $$I_{n}=P_{Tx}+G_{Tx}+G_{Rx}-P_{loss}-L$$

In Equation  (1), $I_{n}$ is the interference power, $P_{Tx}$ represents the transmission power, $G_{Tx}$ represents the gain of the *Tx* antenna, $G_{Rx}$ represents the gain of the *Rx* antenna, $P_{loss}$ is the path loss, and *L* is other losses.

$P_{Tx}$ and $G_{Tx}$ primarily depend on the type of base station and antenna. Models provided in ref. [28] are mainly applicable to urban scenarios and not suitable for aircraft operation scenarios. In the calculation of vertical prohibited zones, the protection radius may vary with flight altitude. Aircraft may be subjected to radiation interference from base stations at higher altitudes. Therefore, it is necessary to consider the architectural and topographical factors near airports. $G_{Rx}$ reflects the impact of aircraft attitude changes on interference. The radio altimeter antenna typically has maximum gain in the direction perpendicular to the aircraft body. When the aircraft pitches or rolls, the direction of maximum gain changes, making it easier for interference to enter the radio altimeter.

#### 2.1.1. 5G Base Station Antenna

Modern 5G base stations commonly utilize beamforming technology, which enhances the directional properties of the beams. There are two types of antennas, namely omnidirectional antennas and array antennas [29]. Array antennas come in various forms, such as 4 × 4, 8 × 8, and 16 × 16. Omnidirectional antennas have equal gain in all directions and lack directional properties. Among the array antennas, a 16 × 16 beamforming antenna has the strongest directional characteristics. Considering the practical interference scenarios, we will model and analyze both an omnidirectional antenna and a 16 × 16 beamforming antenna.

**Figure 4:**
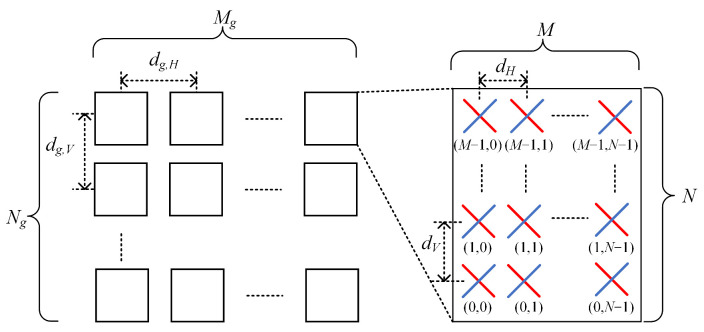
Beamforming antenna.

A beamforming antenna employs a rectangular antenna array configuration to achieve optimal performance. As shown in Figure 4, the array is composed of $M_{g}\times N_{g}$ panels, with $M_{g}$ denoting the number of panels in a column and $N_{g}$ indicating the number of panels in a row. In an effort to maximize the antenna’s efficiency, the antenna panels are uniformly spaced both horizontally and vertically, with spacings of $d_{g,H}$ and $d_{g,V}$, respectively. On each antenna panel, an array of antenna elements is strategically placed in both the vertical and horizontal directions to ensure the greatest possible signal gain. The number of columns is denoted by *N*, while the number of antenna elements in each column with the same polarization is represented by *M*. The antenna elements themselves are also uniformly spaced, with a horizontal spacing of $d_{H}$ and a vertical spacing of $d_{V}$.

**Figure 5:**
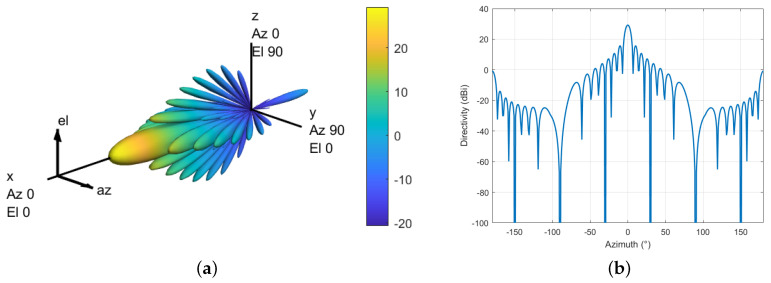
Beamforming antenna pattern. (**a**) 3D. (**b**) Elevation angle θ=0°.

Figure 5a,b illustrate the radiation pattern of a 16 × 16 beamforming antenna. In the main lobe direction, the maximum gain can reach 29.5 dBi, and multiple side lobes appear in both the horizontal and vertical directions. With the utilization of beamforming technology, the direction of the main lobe and side lobes can be adjusted within a certain range. According to ref. [30], the maximum vertical scan angle is ±10°. During interference analysis, the interference from both the main lobe and the side lobes needs to be considered [24].

#### 2.1.2. RA Antenna

The RA antenna is the primary coupling path for 5G interference [3]. It is typically a microstrip patch antenna, and the antenna pattern can be described as Equation (2) [1].

(2) $$G_{RA,dB}\left(\varphi \right)=-\frac{12}{{\varphi_{3dB}}^{2}}\varphi^{2}+G_{RA,dBi}$$

In Equation (2) $G_{RA,dB}\left(\varphi \right)$ is the antenna gain at the azimuth angle (φ), $\varphi_{3dB}$ is 3 dB lobe width of the radio altimeter antenna, and $G_{RA,dBi}$ is the maximum gain of the radio altimeter antenna.

**Figure 6:**
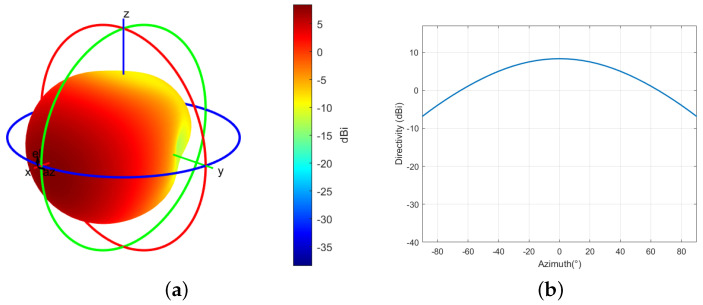
RA antenna pattern. (**a**) 3D. (**b**) Elevation angle θ=0°.

As shown in Figure 6a,b the radio altimeter antenna offers a gain of 2–8 dBi and a coverage angle of 35–60° to the 3 dB point of the antenna pattern. Such wide antenna beams are imperative due to the wide range of pitch and roll angles that an aircraft can reach in flight. Regrettably, radio altimeter antennas are inherently exposed to numerous interference sources, including 5G BSs. Since the antennas are pointed towards the Earth’s surface, they are susceptible to all possible interference sources that may be encountered during operation.

#### 2.1.3. Path Loss

**Figure 7:**
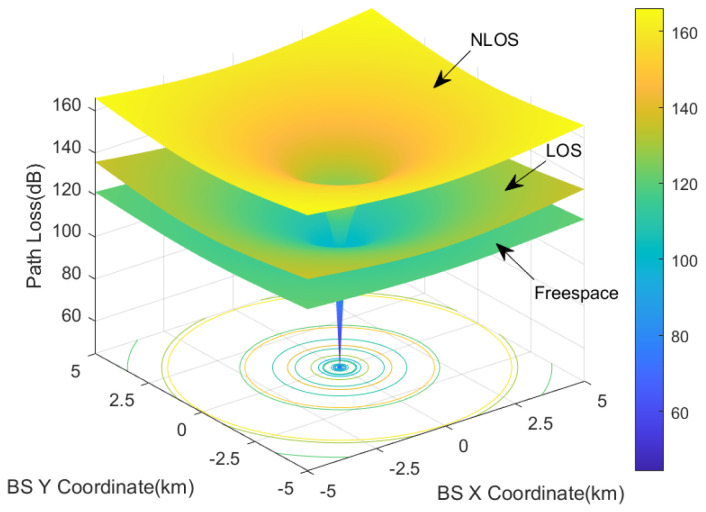
Path loss of free space, LOS, and NLOS.

In actual operational scenarios, path loss ($P_{loss}$) should include both Line-of-Sight (LOS) and Non-Line-of-Sight (NLOS) conditions [30]. LOS indicates that there is a direct line of sight between the interference source and the affected object, without any obstacles. NLOS indicates that there is no direct line of sight due to obstacles.The computed results for the path loss in free-space, line-of-sight (LOS), and non-line-of-sight (NLOS) scenarios are presented in Figure 7.

The conditions for calculating the path loss in Figure 7 include a frequency of 3.93 GHz, a RMa base station height of 35 m, a building height of 7 m, a street width of 25 m, and a height of 1 m for the target object. At a distance of 5 km, the path loss value obtained in the LOS scenario can be approximately 15 dB higher than that in free space, while the path loss value obtained in the NLOS scenario can be approximately 23 dB higher than that in free space. Figure 7 also illustrates the changing trends of the three types of losses with distance. At shorter distances, the attenuation increases more rapidly, while at longer distances, the increase in attenuation becomes more gradual. In the deployment protection method, we assume that the path loss between the RA antenna and the base station is random, with a 50% probability of LOS loss and a 50% probability of NLOS loss.

### 2.2. Interference Thresholds

From an aviation safety perspective, when analyzing interference with radio altimeters, it is common to consider the worst-case scenario by using the lowest interference threshold. The interference threshold of a radio altimeter can be influenced by multiple factors, including the type of altimeter, altitude AGL, the operational scenario, the safety threshold margin, and more. By considering the worst conditions and using the lowest interference threshold, the analysis ensures that the radio altimeter is adequately protected against interference. In this paper, we primarily focus on the interference caused by fundamental 5G emissions and do not consider the interference threshold caused by spurious 5G emissions. The interference thresholds are shown in Table 2 [2].

First, radio altimeters are divided into the following three categories: category 1, commercial air transport aircraft; category 2, regional, business aviation, and general aviation aircraft; and category 3, helicopters. In this study, we focus on category 2 radio altimeters to establish interference thresholds. This is because category 2 has the lowest interference threshold among the three categories, as indicated in Table 2, resulting in the largest prohibited zone [31].

Second, because the distance measurement echo power changes with the flight altitude, the sensitivity of modern radio altimeters is generally designed to be adjustable according to the altitude. Therefore, the interference threshold of the radio altimeter also changes with the flight altitude. When considering the prohibited zone, we should discuss the size of the prohibited zone at different altitudes AGL in detail.

Third, the ICAO *Handbook on Radio Frequency Spectrum Requirements for Civil Aviation* recommends that an aviation safety margin of 6–10 dB be included in the interference analysis concerning aeronautical safety systems in order to address the risk that some factors cannot be foreseen [32]. A safety margin of not less than 6 dB should be applied until it is established on a case-by-case basis. For the radar altimeter’s integrity, availability, and/or continuity, a safety margin of not less than 6 dB must be included in the interference analysis. The occurrence rate of harmful interference is expected to be evaluated on the order of 1 × $10^{-5}$ per flight hour [3]. This occurrence rate is associated with operational scenarios with major failure conditions.

**Figure 8:**
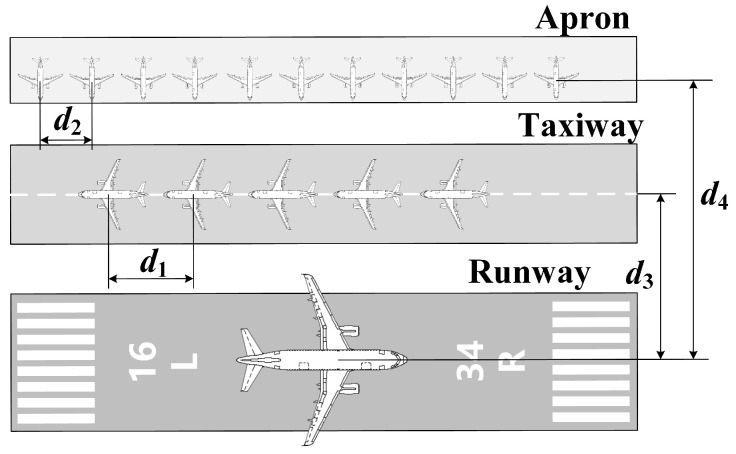
Worst-case landing scenario (WCLS).

Finally, all thresholds are provided based on the worst-case landing scenario (WCLS). Takeoff and approach are the most susceptible phases to interference. For the radio altimeter, it is essential to consider the RA signals generated by other aircraft on the taxiway and apron during these phases. As shown in Figure 8, it is assumed that five aircraft are taxiing on the taxiway parallel to and at a distance of $d_{3}$ from the runway, with a spacing of $d_{1}$. Additionally, eleven aircraft with a spacing of $d_{2}$ are parked on the apron parallel to and at a distance of $d_{4}$ from the runway. At this time, the 16 aircraft outside the runway will also generate interference simultaneously, thus affecting the threshold. Their interferences will be lower compared to the scenario where the WCLS is not considered.

### 2.3. Deployment Protection Methods

The deployment protection methods proposed in this paper extend the aforementioned approach by conducting interference analysis for all base station positions within a designated zone surrounding the aircraft. They entail an integrated model that incorporates all base station parameters, as well as flight dynamic parameters. Through this analysis, the prohibited zone for base station deployment is determined. The prohibited zone is not only horizontal but also vertical. The size of the prohibited zone varies according to various factors, such as the type of path loss, antenna parameters, radio altimeter type, interference thresholds, flight altitude and attitude, and base station parameters. Additionally, the deployment protection methods include interference mitigation methods. With the use of beamforming technology, base stations can reduce interference with radio altimeters by implementing power control and angle shutoff techniques.

#### 2.3.1. Prohibited Zone

The prohibited zone is obtained by following the procedure shown in Algorithm 1. First, the aircraft is located at point (0,0,H), where *H* represents the altitude AGL, with this paper considering altitudes of 100 ft, 1000 ft, and 2000 ft. Second, within a three-dimensional space of dimensions (X,Y,H), the antenna gain ($G_{bf}$) of the base station at a coordinates (x,y,h) is computed. Due to the use of beamforming technology, $G_{bf}$ is not only dependent on the coordinates (x,y,h) but also on the vertical scan angle (θ). Additionally, the  gain ($G_{a}$) of the radio altimeter antenna in this direction is related to the coordinates (x,y,h) and the pitch\rollangles of the aircraft α\β. When considering path loss ($P_{loss}$), a random function is employed to ensure that the probabilities of line-of-sight loss ($P_{LOS}$) and non-line-of-sight loss ($P_{NLOS}$) occurring are both 50%. Third, the interference with the aircraft (I(x,y,h)) generated by the base station at the coordinates (x,y,h) is calculated. When I(x,y,h) equals the interference threshold value (Th), the coordinates of this point are recorded as $z_{i}$. All points are traversed to find all $z_{i}$ values. Finally, all points are connected to form the outline of the entire prohibited zone. Within the outline, a base station may cause interference with the aircraft. Outside the outline, interference is below the interference threshold.
**Algorithm 1** Calculation method for the prohibited zone**Input:** (X,Y,H)**Output:** Outline  1:**repeat**  2:    **repeat**  3:        **repeat**  4:           $G_{Tx}=G_{bf}\left(x,y,h,\theta \right)$  5:           $G_{Rx}=G_{a}\left(x,y,h,\alpha \backslash \beta \right)$  6:           $P_{loss}=random\left(P_{LOS},P_{NLOS}\right)$  7:           $I\left(x,y,h\right)=P_{Tx}+G_{Tx}+G_{Rx}-P_{loss}$  8:           **if** I(x,y,h)=Th **then** $z_{i}=\left(x,y,h\right)$  9:               i=i+110:           **else**11:               i=i12:           **end if**13:           x=x+114:        **until** x=X15:        y=y+116:    **until** y=Y17:    h=h+118:**until** h=H19:$Outline=\{z_{1},z_{2},\ldots ,z_{i-1}\}$

The interference threshold varies at different altitudes AGL. For example, at 2000 ft, the interference threshold can be as low as −69 dBm.The attenuation caused by increasing altitude cannot compensate for the decrease in the interference threshold, resulting in a larger prohibited zone. The pitch\roll angle of the aircraft also causes a change in the gain of the altimeter antenna. It provides a larger gain for the interfering signal, which can make the prohibited zone larger.

In the context of aircraft operation, the prohibited zones extend not only to the horizontal plane but also to the vertical plane. The horizontal prohibited zones imply that base stations located within the horizontal vicinity of the runway and around the aircraft’s ground projection must be prohibited. The vertical prohibited zones refer to the fact that base stations situated atop tall buildings, mountains, and undulating terrain must also be prohibited.

#### 2.3.2. Interference Mitigation Methods

Interference mitigation methods can help reduce the size of the prohibited zone by implementing technical measures during base station deployment to minimize interference with radio altimeters. These methods involve employing techniques such as power control and angle shutoff using beamforming technology. By adjusting the power levels and directing the beams appropriately, interference with radio altimeters can be reduced [26]. We propose two methods for interference mitigation.

Algorithm 2 illustrates the steps involved in the power control method. Within the prohibited zone, a total of *N* points are unsuitable for base station deployment, with all base station coordinates recorded in $BS_{list}$. First, the transmit power ($P_{i}$) of the *i*-th base station is reduced by 1 dB. Then, a calculation is performed to determine whether the interference ($I_{i}$) generated by this base station is less than the interference threshold (Th). If so, the counter is incremented using $q_{j}$. This process is repeated until $P_{i}<0$. Second, calculations are performed for all base stations in $BS_{list}$ to obtain the proportion ($P_{TxCon}$) of base stations with power reduction values of 1 dB, 2 dB, …, and j dB using $\frac{q_{1}}{N},\frac{q_{2}}{N},\ldots ,\frac{q_{j}}{N}$.
**Algorithm 2** Power Control Method**Input:** $BS_{list},N$**Output:** $P_{TxCon}$  1:**repeat**  2:    **repeat**  3:        $P_{i}=P_{i}-1$  4:        **if** $I_{i}<Th$ **then** $q_{j}=q_{j}+1$  5:           j=j+1  6:        **else**  7:           $q_{j}=q_{j}$  8:        **end if**  9:    **until** $P_{i}<0$10:    i=i+111:**until** i=N12:$P_{TxCon}=\left\{\frac{q_{1}}{N},\frac{q_{2}}{N},\ldots ,\frac{q_{j}}{N}\right\}$

**Figure 9:**
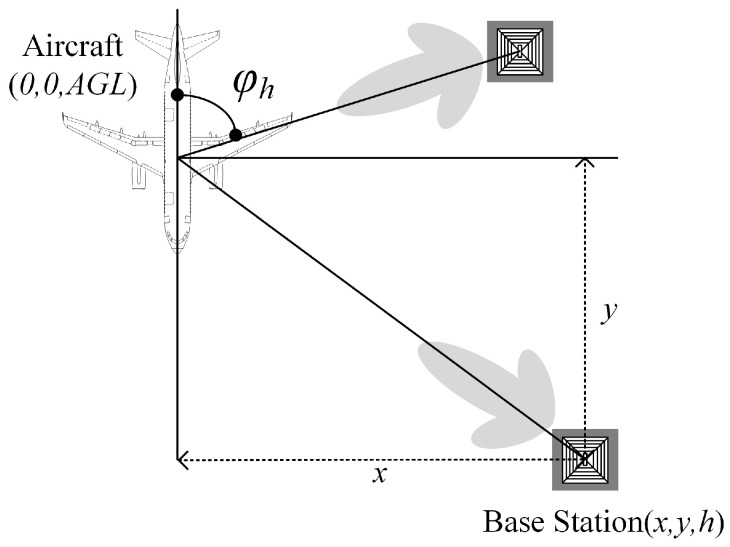
Top view of interference scenario.

**Figure 10:**
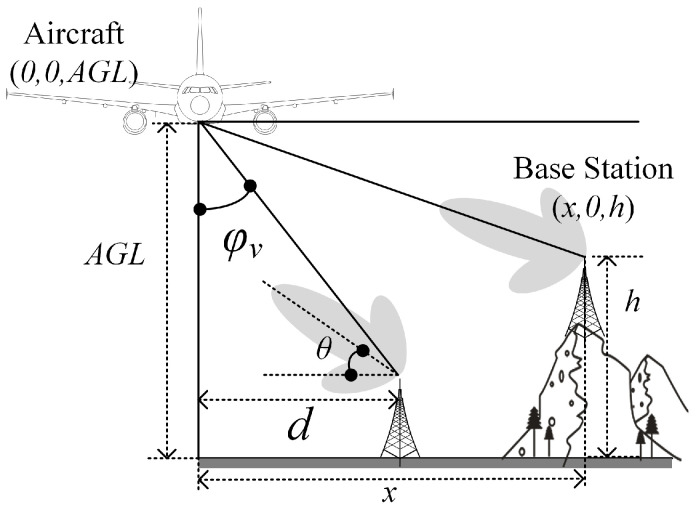
Front view of interference scenario.

Algorithm 3 presents the angle shutoff method, which takes advantage of the benefits of beamforming technology. In the context of interference mitigation, it is essential to analyze the angles at which the beams may cause interference and selectively them off. In the horizontal plane, the angle $\varphi_{h}$ in Figure 9 is from 1° to 360°. In the vertical plane, $\varphi_{v}$ in Figure 10 is from 0° to 90°. Algorithm 3 is applicable to both $\varphi_{v}$ and $\varphi_{h}$ but with different calculation ranges. First, $\varphi_{v}$ or $\varphi_{h}$ is initialized at 0, and the horizontal distance (*d*) between the base station and the aircraft is defined, as shown in Figure 10. Then, a calculation is performed to determine whether the interference ($I(\varphi_{h}\backslash \varphi_{v})$) generated by the base station is less than the interference threshold (Th). If so, the counter is incremented using $\varphi_{off}\left(\varphi_{h}\backslash \varphi_{v}\right)$. This process is repeated to obtain the proportion ($An_{Shuth}$) of base stations with $\varphi_{off}\left(\varphi_{h}\right)$ values ranging from 1° to 360° using $\frac{\varphi_{off}\left(1\right)}{360},\frac{\varphi_{off}\left(2\right)}{360},\ldots ,\frac{\varphi_{off}\left(360\right)}{360}$ to similarly obtain $An_{Shutv}$.
**Algorithm 3** Angle Shutoff Method**Input:** $\varphi_{h}\backslash \varphi_{v}=0,d$**Output:** $An_{Shuth}\backslash An_{Shutv}$  1:**repeat**  2:    **if** $I(\varphi_{h}\backslash \varphi_{v})>Th$ **then** $\varphi_{off}\left(\varphi_{h}\backslash \varphi_{v}\right)=\varphi_{off}\left(\varphi_{h}\backslash \varphi_{v}\right)+1$  3:        $\varphi_{h}\backslash \varphi_{v}=\varphi_{h}\backslash \varphi_{v}+1$  4:    **else**  5:        $\varphi_{off}\left(\varphi_{h}\backslash \varphi_{v}\right)=\varphi_{off}\left(\varphi_{h}\backslash \varphi_{v}\right)$  6:    **end if**  7:**until** $\varphi_{h}=360$ \$\varphi_{v}=90$  8:$An_{Shuth}=\left\{\frac{\varphi_{off}\left(1\right)}{360},\frac{\varphi_{off}\left(2\right)}{360},\ldots ,\frac{\varphi_{off}\left(360\right)}{360}\right\}$ \$An_{Shutv}=\left\{\frac{\varphi_{off}\left(1\right)}{90},\frac{\varphi_{off}\left(2\right)}{90},\ldots ,\frac{\varphi_{off}\left(90\right)}{90}\right\}$

**Figure 11:**
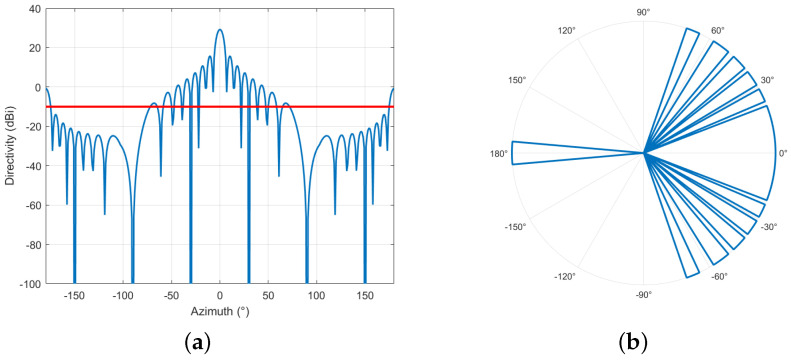
Angle shutoff. (**a**) Angle shutoff in Cartesian coordinate system. (**b**) Angle shutoff in polar coordinate system.

To better understand the concept of angle shutoff, we represented it in both Cartesian and polar coordinate systems, as shown in Figure 11a,b. Figure 11a illustrates the relationship between angle shutoff and azimuth. In the antenna pattern (Cartesian coordinates) of the 16 × 16 beamforming antenna, assuming the interference threshold is −10 dB (the red line), the angles above the interference threshold need to be shut off. Figure 11b illustrates the angles that need to be shut off in the polar coordinate system. The angle shutoff is the sum of all the angles that exceed the interference threshold. It represents the total angular range that needs to be shut off in order to mitigate interference. The angle shutoff can be obtained by summing up the individual angles that exceed the interference threshold within the specified range. By calculating the angle shutoff, we can determine the extent of interference mitigation required to minimize the impact on radio altimeters.

## 3. Simulation Parameters

The top and front views of the interference scenario are shown in Figure 9 and Figure 10. The aircraft’s position coordinates are (0,0,AGL), where AGL refers to altitude above ground level. The base station’s position coordinates are (x,y,h). The angle between the aircraft and the base station in the top view is denoted by $\varphi_{h}\in \left(0,360{}^{\circ}\right)$. Similarly, in the front view, the angle between the aircraft and the base station is defined as $\varphi_{v}\in \left(0,90{}^{\circ}\right)$. For computational simplicity, the base station’s coordinates in the front view can be expressed as (x,0,h). Additionally, the horizontal distance (*d*) between the base station and the aircraft is defined for mitigation measure calculations.

The base station parameters are shown in Table 3 based on the 3GPP standards [33,34]. There are three types of base stations, namely RMa, UMa, and UMi stations. Among them, an RMa station has the highest transmission power and the highest base station height. In terms of antenna types, all three types of base stations can use a 15 dBi omnidirectional antenna and a 16 × 16 beamforming antenna with a maximum gain of 29.5 dBi. In the RMa scenario, we assume a building height of 7 m and a street width of 25 m. For the calculation of path loss, shadow fading values are assumed for all three types of base station. Additionally, the maximum vertical scan angle for all base stations is 10°.

## 4. Results and Discussion

### 4.1. Prohibited Zone with Omnidirectional Antenna

The prohibited zones encompass both the horizontal and vertical dimensions. The horizontal prohibited zones are defined by points in the XOY (horizontal) plane that exceed the interference threshold. The vertical prohibited zones are defined by points in the XOZ (vertical) plane that exceed the interference threshold. We consider these prohibited zones for both the omnidirectional antenna and the 16 × 16 beamforming antenna configurations.

Initially, we assume a flight altitude of 200 ft for the aircraft and subsequently analyze the scenarios at 1000 ft and 2000 ft. In Table 2, category 2 altimeters are exclusively applicable for altitudes below 2000 ft and are most susceptible to interference. Hence, only interference scenarios below 2000 ft are considered. As for the category 1 altimeters in Table 2, the change in interference threshold relative to 2000 ft is inconspicuous at 5000 ft. However, path loss significantly increases at 5000 ft. Therefore, it is evident that the prohibited zone at 5000 ft is smaller than at 2000 ft. Consequently, conducting analyses for altitudes of 200 ft, 1000 ft, and 2000 ft effectively encompasses typical application scenarios.

#### 4.1.1. Horizontal Prohibited Zone

**Figure 12:**
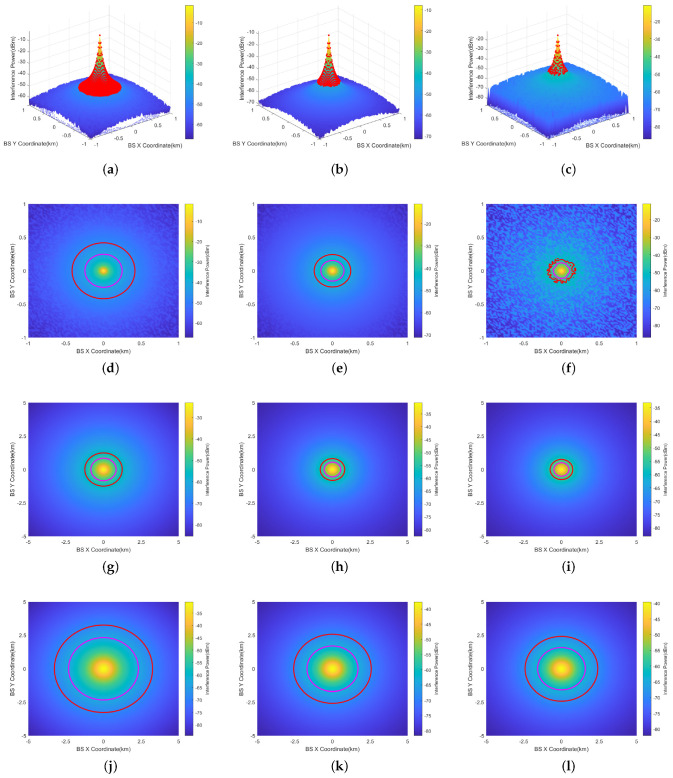
Horizontal Prohibited zones of RMa, UMa, and UMi at AGL = 200 ft, 1000 ft, 2000 ft (omnidirectional antenna). (**a**) RMa (AGL=200 ft, 3D). (**b**) UMa (AGL=200 ft, 3D). (**c**) UMi (AGL=200 ft, 3D). (**d**) RMa (AGL=200 ft). (**e**) UMa (AGL=200 ft). (**f**) UMi (AGL=200 ft). (**g**) RMa (AGL=1000 ft). (**h**) UMa (AGL=1000 ft). (**i**) UMi (AGL=1000 ft). (**j**) RMa (AGL=2000 ft). (**k**) UMa (AGL=2000 ft). (**l**) UMi (AGL=2000 ft).

Figure 12a–c illustrate the 3D representations when the aircraft’s altitude AGL is 200 ft (60.96 m) for RMa, UMa, and UMi scenarios, respectively. The three-dimensional coordinate system showcases the *X*-axis, representing the base station’s *X*-coordinate on the horizontal plane, and the *Y*-axis, representing the base station’s *Y*-coordinate on the horizontal plane. The *Z*-axis indicates the level of interference detected at the aircraft’s position, depicted through color differentiation.

To elaborate further on the concept of the prohibited zone, 2D representations of 200 ft for RMa, UMa, and UMi scenarios are also provided in Figure 12d–f. The results are calculated within ±1 km. The regular and irregular red circles in the figures are prohibited zones. Base stations exceeding the interference threshold are distributed within the zone enclosed by the red line. Outside the red line, the interference power attenuates due to increased distance, falling below the interference threshold. Two-dimensional representations of 1000 ft (304.8 m) are provided in Figure 12g–i, and representations of 2000 ft (609.6 m) are provided in Figure 12j–l. The results are calculated within ±5 km.

- Among the three types of base station, the prohibited zone of the RMa base station is the largest. This is primarily because the RMa base station has the highest transmission power and height.
- For the same type of base station, the prohibited zone is largest when AGL=2000 ft and smallest when AGL=200 ft. This is the result of the dynamic variation of both interference threshold and path loss.
- The magenta line represents the prohibited zone without considering the 6 dB margin. The ICAO 6 dB margin increases the prohibited zone radius by 0.2–0.9 km.
- The irregularity of the prohibited zones in Figure 12f is primarily attributed to the statistical feature of line-of-sight (LOS) and non-line-of-sight (NLOS) path loss, assuming that the occurrence probabilities of both LOS and NLOS are equal to 50%. In UMi scenario, there is a significant difference in the attenuation between LOS and NLOS due to the fact that the UMi base station has a height of 10 m, which is the lowest among the three types of base stations. This results in a greater difference in attenuation between NLOS and LOS. In Figure 12i,l, with AGL=1000 ft, 2000 ft, the aircraft is at a higher altitude. The path loss is nearly identical between the LOS and NLOS scenarios, and the statistical feature of path loss is not pronounced. As a result, the prohibited zone does not exhibit irregularity.

#### 4.1.2. Vertical Prohibited Zone

The vertical prohibited zone is a crucial aspect that is often disregarded. However, a base station erected on a mountain has the potential to radiate to aircraft in the sky. To simplify the analysis, assuming the RA antenna gain is symmetrical, only the vertical plane needs to be studied.

**Figure 13:**
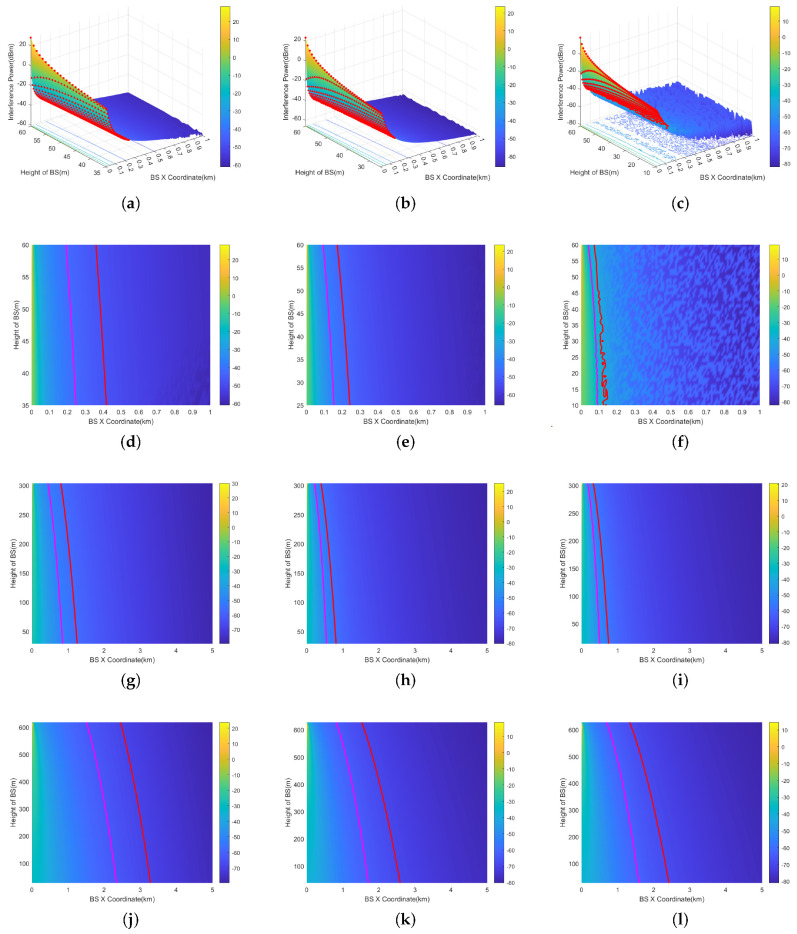
Vertical prohibited zones of RMa, UMa, and UMi at AGL = 200 ft, 1000 ft, 2000 ft (omnidirectional antenna). (**a**) RMa (AGL=200 ft, 3D). (**b**) UMa (AGL=200 ft, 3D). (**c**) UMi (AGL=200 ft, 3D). (**d**) RMa (AGL=200 ft). (**e**) UMa (AGL=200 ft). (**f**) UMi (AGL=200 ft). (**g**) RMa (AGL=1000 ft). (**h**) UMa (AGL=1000 ft). (**i**) UMi (AGL=1000 ft). (**j**) RMa (AGL=2000 ft). (**k**) UMa (AGL=2000 ft). (**l**) UMi (AGL=2000 ft).

The 3D results of the 200 ft (60.96 m) scenario is depicted in Figure 13a,b. The X-axis represents the base station’s *X*-coordinate, and the *Y*-axis signifies the height of the BSs. The *Z*-axis indicates the interference level at the aircraft position, depicted through color differentiation. Two-dimensional results are also provided in Figure 13d–f. The results are calculated within 1 km. Two-dimensional representations of 1000 ft (304.8 m) are provided in Figure 13g–i, and representation of 2000 ft (609.6 m) are provided in Figure 13j–l. The results are calculated within 5 km.

- The radius of the vertical prohibited zone varies with height. This is mainly due to the fact that the gain of the RA antenna varies at different angles. Its ability to receive interference is also different. At higher altitudes close to the aircraft’s altitude AGL, the angle ($\varphi_{v}$) in Figure 10 is bigger, and the gain decreases accordingly.
- Similar to the horizontal prohibited zone, the largest vertical prohibited zone occurs with the RMa base station at 2000 ft. The magenta line represents the ICAO 6 dB margin, which increases the prohibited zone radius by 0.2–1.1 km. The path loss of NLOS and LOS is the most significant in the UMi scenario.

### 4.2. Prohibited Zone with Beamforming Antenna

Herein, we focus on a beamforming antenna. A beamforming antenna has a main lobe and several side lobes. The antenna gain is significantly different in at different angles. In order to better consider a real-world interference scenario, the following assumptions are made for the beamforming antenna:

(1) A commonly used 16 × 16 beamforming antenna is selected.
(2) The main lobe of the beamforming antenna can sweep in a 360° range horizontally using electronic steering technology.
(3) The main lobe of the beamforming antenna can sweep in range of 0–10° vertically. The angle between the main lobe and the horizontal plane is the vertical scan angle (θ) (θ∈(0,10°)), as shown in Figure 10.

Furthermore, based on the analysis results of the omnidirectional antenna, the RMa base station has the largest prohibited zone. In order to simplify the analysis process, only the RMa base station is discussed in detail in the analysis.

#### 4.2.1. Horizontal Prohibited Zone

**Figure 14:**
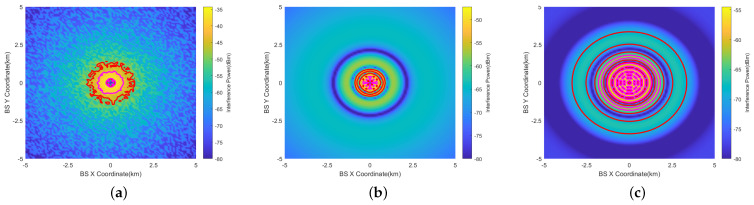
Horizontal prohibited zones of RMa at AGL = 200 ft, 1000 ft, 2000 ft (beamforming antenna (θ=0°)). (**a**) AGL=200 ft. (**b**) AGL=1000 ft. (**c**) AGL=2000 ft.

Figure 14 illustrates the horizontal prohibited zones for the RMa base station using the 16 × 16 beamforming antenna at θ=0°.

- The largest prohibited zone occurs at AGL=2000 ft. The prohibited zone at AGL=1000 ft is smaller than that at AGL=200 ft. This is because at 1000 ft, the interference is mainly generated by the side lobes of the antenna.
- Within the prohibited zone (the largest red circle), there are still multiple smaller red circles. This is most evident in Figure 14c. This occurs because there are angles with minimal gain between different lobes of the beamforming antenna. At these angles, the interference is minimal. These smaller circles should be disregarded when determining the size of the prohibited zone.
- The prohibited zone of RMa exhibits irregular characteristics when AGL=200 ft. The statistical features of LOS and NLOS can manifest at the edges of the prohibited zone. However, these features are not evident when AGL=1000 ft at 2000 ft.

**Figure 15:**
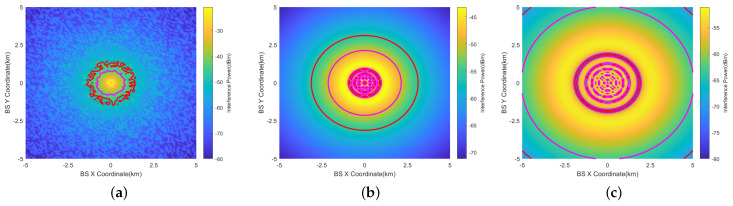
Horizontal prohibited zones of RMa at AGL=200 ft, 1000 ft, 2000 ft (beamforming antenna (θ=10°)). (**a**) AGL=200 ft. (**b**) AGL=1000 ft. (**c**) AGL=2000 ft.

Figure 15 depicts the prohibited zone when the vertical scan angle of the beamforming antenna is set to 10° (θ=10°). At 200 ft, θ has a negligible impact on the prohibited zone. At 1000 ft, the receiving antenna (RA) captures the radiated energy of the main beam, resulting in a larger prohibited zone. At 2000 ft, the prohibited zone is the largest, with a radius of 6.7 km.

#### 4.2.2. Vertical Prohibited Zone

**Figure 16:**
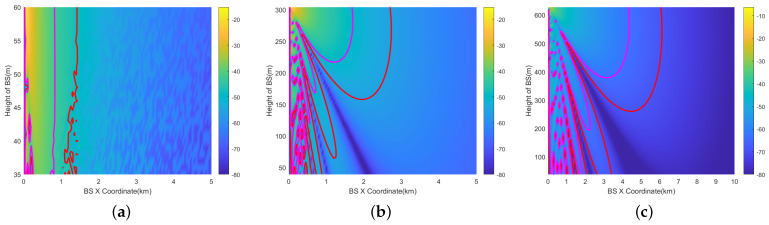
Vertical prohibited zones of RMa at AGL = 200 ft, 1000 ft, 2000 ft (beamforming antenna (θ=0°)). (**a**) AGL=200 ft. (**b**) AGL=1000 ft. (**c**) AGL=2000 ft.

Figure 16 illustrates the vertical prohibited zones for the RMa base station using the 16 × 16 beamforming antenna at θ=0°.

- The largest prohibited zone occurs at AGL=2000 ft. At 200 ft (60.96 m), the radius of the prohibited zone shows little variation within the 0–200 ft height range. At 1000 ft (304.6 m), the radius of the vertical prohibited zone exhibits a significant change, ranging from 0.9 km to 2.8 km. Due to interference from side lobes, the shape of the prohibited zone becomes complex. At 2000 ft (609.6 m), the shape of the prohibited zone is similar to 1000 ft, varying from 3.4 km to 6.1 km.
- This implies that even when the aircraft reaches flight altitudes of 1000 ft or 2000 ft, stations on the mountain located as far as 2.8 km and 6.1 km away can still cause interference. However, base stations on the ground do not cause interference.

**Figure 17:**
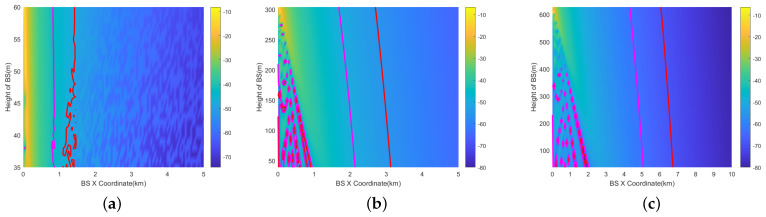
Vertical Prohibited zones of RMa at AGL = 200 ft, 1000 ft, 2000 ft (beamforming antenna (θ=10°)). (**a**) AGL=200 ft. (**b**) AGL=1000 ft. (**c**) AGL=2000 ft.

In Figure 17, the prohibited zones are presented for a vertical scan angle (θ) of the beamforming antenna set to 10°.

- At 200 ft, the impact of θ is minimal. At 1000 ft, there is a notable change in the shape of the vertical prohibited zone, primarily due to the aircraft being within the main lobe range of the antenna. At 2000 ft, the shape of the vertical prohibited zone is similar to that at 1000 feet, with a range of change from 6.2 km to 6.7 km.

### 4.3. Effect of Aircraft Pitch\Roll Angle

The interference acceptance angle of the RA antenna is affected by the pitch\rollangle of the aircraft, which influences the size of the prohibited zone. We define pitch\roll angle of the aircraft as α\β. The depicted scenarios assume that the RA antenna exhibits axisymmetric characteristics. Hence, the effects of a 30° pitch (α=30°) and a 30° roll (β=30°) can be considered almost identical.

**Figure 18:**
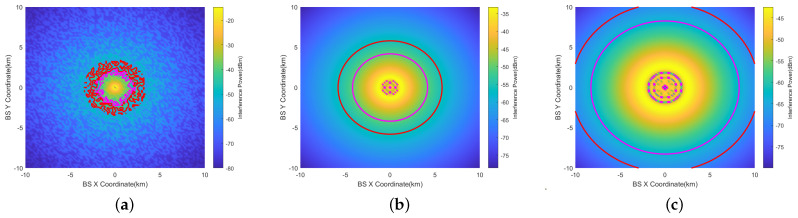
Horizontal prohibited zones of RMa at AGL = 200 ft, 1000 ft, 2000 ft (beamforming antenna (θ=0°, α\β=30°)). (**a**) AGL=200 ft. (**b**) AGL=1000 ft. (**c**) AGL=2000 ft.

**Figure 19:**
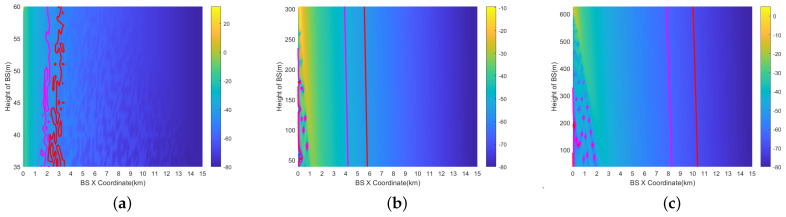
Vertical prohibited zones of RMa at AGL = 200 ft, 1000 ft, 2000 ft (beamforming antenna (θ=10°, α\β=30°)). (**a**) AGL=200 ft. (**b**) AGL=1000 ft. (**c**) AGL=2000 ft.

Civil aviation aircraft are unlikely to reach ±30° at an altitude of 200 ft. However, assuming a maximum of ±30° means the worst-case interference scenario. Figure 18 and Figure 19 provide the results of the prohibited zone with a vertical scan angle of 10° (θ=10°) and aircraft pitch\roll angles of ±30°(α\β=30°). The horizontal results are calculated within ±10 km. The vertical results are calculated within 15 km. The horizontal and vertical prohibited zones significantly increase at all three altitudes AGL.

### 4.4. Interference Mitigation Methods in Prohibited Zone

To optimize the deployment of the base station and reduce interference with the RA, two methods were analyzed, namely Tx power control and angle shutoff. These methods were studied in the scenario of the largest prohibited zone, with RMa BSs and AGL=2000 ft for the omnidirectional antenna and the beamforming antenna.

#### 4.4.1. Tx Power Control

**Figure 20:**
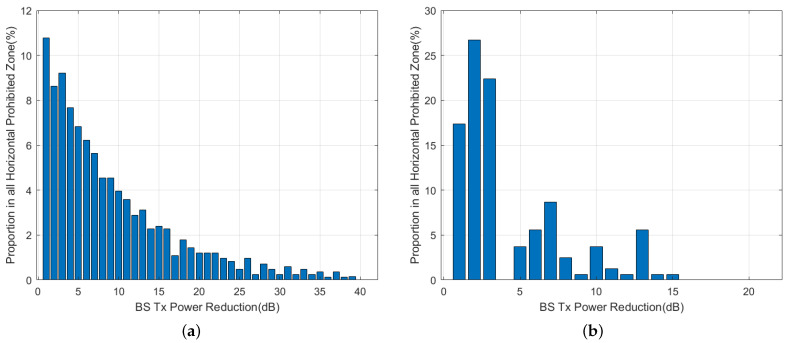
Tx power control in horizontal prohibited zone. (**a**) Omni, 2000 ft. (**b**) 16 × 16 Beamforming, 2000 ft, θ=0°, α\β=0°.

**Figure 21:**
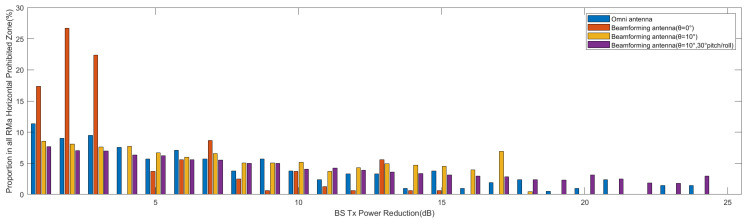
Tx power control in horizontal prohibited zone for different scenarios.

In Figure 20a,b, the results of horizontal prohibited zones for both omnidirectional and beamforming antennas are shown for a vertical scan angle of θ=0° and an aircraft pitch/roll angle of α\β=0°. The results show that for omnidirectional antennas, the base stations require up to a 40 dB attenuation of the Tx power, and 10.8% of the base stations require a 1 dB attenuation, which is the highest percentage. For beamforming antennas, the maximum required attenuation is 15 dB, and 27% of the base stations require a 2 dB attenuation, which is the highest percentage. This implies that Tx power reduction is more effective for beamforming antennas. Furthermore, there is a significant difference in results between the use of omnidirectional and beamforming antennas due to the directional attenuation produced by beamforming antennas. Figure 21 shows a comparison of attenuation within a range of 25 dB for four different scenarios. The power attenuation ratio for the base stations varies significantly for different scenarios.

**Figure 22:**
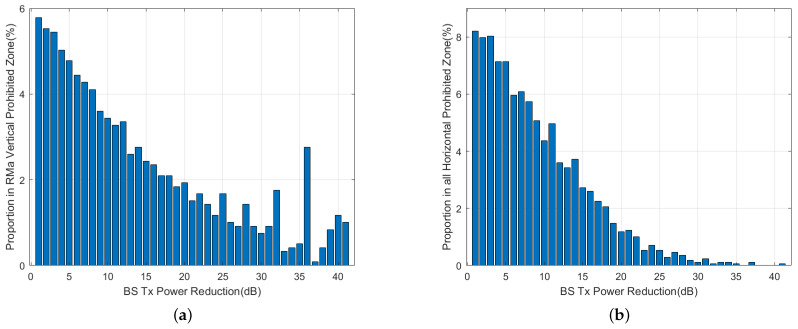
Tx power control in vertical prohibited zone (16 × 16 beamforming, 2000 ft, θ=0°, α\β=0°). (**a**) Omni, 2000 ft. (**b**) 16 × 16 Beamforming, 2000 ft, θ=0°, α\β=0°.

**Figure 23:**
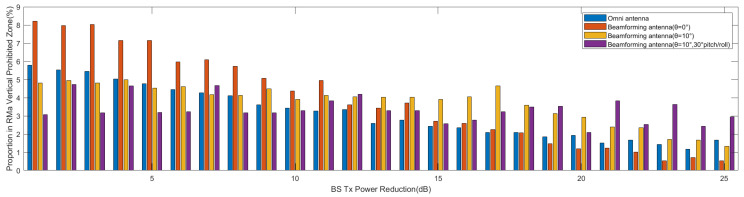
Tx power control in vertical prohibited zone for different scenarios.

The results of vertical prohibited zones for both omnidirectional and beamforming antennas are shown in Figure 22a,b for a vertical scan angle of θ=0° and an aircraft pitch and roll angle of α\β=0°. For the vertical prohibited zone, analysis of the 40 dB attenuation range can cover most of the base stations. The proportion of base stations that require 1 dB attenuation ranges between 5.8% and 8.1%, which is the highest. The proportion of base stations in the four scenarios within the 25 dB attenuation range is compared in Figure 23, and as the attenuation level increases, the percentage of corresponding base stations shows a downward trend overall.

#### 4.4.2. Angle Shutoff Method

The angle shutoff method is only suitable for beamforming antennas. It needs to be carried out separately for the horizontal and vertical prohibited zones. In the horizontal prohibited zone, assuming that the main lobe of the beamforming antenna can sweep through a 360° range horizontally, the horizontal distance between the base station and the the aircraft is the main factor that affects angle shutoff. In the vertical prohibited zone, the horizontal distances between the base station and the aircraft are 1 km, 3 km, and 5 km. Then, the relationship between the angle shutoff and $\varphi_{v}\in \left(0,90{}^{\circ}\right)$ is analyzed. Figure 10 shows the definition of the horizontal distance (*d*) and the definition of $\varphi_{v}$. The following assumptions are made:

(1) The horizontal distance between the aircraft and BS varies between 0 and 10 km;
(2) The steering beam of the BS varies between 0 and 360° horizontally;
(3) All possible directions of the beamforming antenna of the base station need to be analyzed;
(4) All angles of interference at different altitudes AGL need to be analyzed.

**Figure 24:**
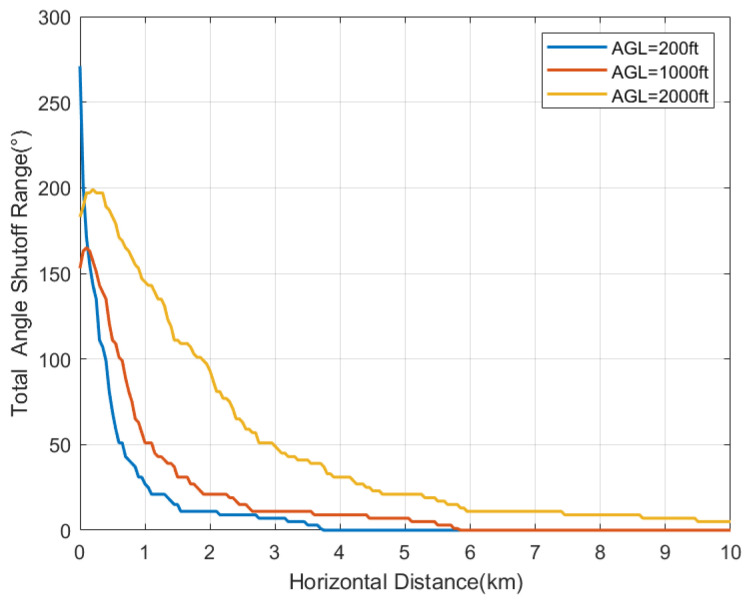
Relationship between angle shutoff and *d*.

Figure 24 illustrates the relationship between the angle shutoff and the horizontal distance (*d*) under three altitude AGL conditions. The angle shutoff decreases gradually as the distance increases. For AGL=200 ft, the angle shutoff may reach up to 270° at close range, while it decreases to 25° at a distance of 1 km. For AGL=1000 ft, the angle shutoff gradually decreases from around 150°. Similarly, for AGL=2000 ft, the angle shutoff gradually decreases from around 200°.

**Figure 25:**
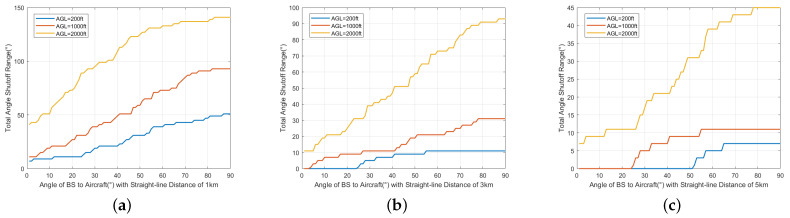
Relationship between angle shutoff and $\varphi_{v}$. (**a**) d=1 km. (**b**) d=3 km. (**c**) d=5 km.

Figure 25a–c illustrate the relationship between the angle shutoff and the $\varphi_{v}$ at different horizontal distances. At the three distances, the angle shutoff increases with increases in $\varphi_{v}$. The greatest angle shutoff is at d=1 km, AGL=200 ft, ranging from 40° to 140°. At d=3 km, the range of the angle shutoff varies from 11° to 93°, while at d=5 km, the angle shutoff varies from 7° to 45°. The larger the $\varphi_{v}$, the stronger the received interference. This is because of the smaller angle between the aircraft and the main lobe of the base station antenna.

### 4.5. Summary

To present the results more clearly, we compare the radius of all prohibited zones in Table 4 and Table 5.

The radius of the horizontal prohibited zone increases with altitude AGL. Without considering the antenna’s vertical scan angle and aircraft attitude, for omnidirectional antennas, *R*_6dB_ increases from 0.4 km to 3.2 km. For beamforming antennas, *R*_6dB_ increases from 1.4 km to 3.4 km. A 10°, the vertical scan angle can increase the protection radius by 0.1 km to 2.5 km. A 30° aircraft pitch\roll angle can further increase the radius by 1.3 km to 3.7 km. Additionally, the ICAO 6 dB also increases the prohibited zone radius by 0.2 km to 2 km.

The radius of the vertical prohibited zone also increases with altitude AGL. In addition to providing the radius on the ground, the radius in the air is also provided. For omnidirectional antennas, the *R*_6dB_ on the ground ranges from 0.2 km to 3.2 km. The *R*_6dB_ in the air ranges from 0.2 km to 2.6 km. For beamforming antennas, the *R*_6dB_ on the ground ranges from 0.8 km to 3.4 km, while the *R*_6dB_ in the air ranges from 0.2 km to 6.1 km. A 10° vertical scan angle can increase the radius by 0.1 km to 2.7 km. A 30° aircraft pitch\roll angle can further increase the radius by 1.3 km to 3.7 km. Additionally, the ICAO 6 dB also increases the prohibited zone radius by 0 km to 2 km.

Regarding the power control method, for omnidirectional antennas, a 1 dB decrease in Tx power within the horizontal prohibited zone increases the deployable base station positions by 10.8%. For beamforming antennas (θ=10°, α\β=30°), a 1 dB decrease in Tx power increases the deployable base station positions by 7.5%. In the vertical prohibited zone, the results are 5.8% and 3.1%. As the Tx power continues to decrease, the incremental increase gradually diminishes.

Regarding the angle shutoff method, under the given distance and angle, most base stations only need to shut off some angles to avoid interference. As the distance increases, the required angle shutoff decreases. At 1 km, it is 140°, reducing to 50° at 3 km and merely 25° at 5 km. However, with an increase in base station height (the increase in $\varphi_{v}$), the required angle shutoff increases at a given distance. It is 145° at 1 km, 93° at 3 km, and 45° at 5 km.

### 4.6. Discussion

#### 4.6.1. The Applicability of the Model

Interference protection models in telecommunications are generally tailored for analyzing interference from base stations on the ground. The adopted interference threshold does not consider aviation safety requirements. The victim systems in these models are typically static ground objects. In contrast, the deployment protection method proposed in this paper integrates typical base station parameters with civil aviation operational scenarios. It comprehensively considers factors such as radio altimeter system characteristics, dynamic interference thresholds, and vertical prohibited zones. This model incorporates key interference parameters into an integrated interference model. It is an interference protection model suitable for the aviation operation and applicable for engineering purposes.

Interference management in the aviation domain has typically relied on spectrum isolation and distance protection. However, within airports, there are still numerous scenarios where 5G is in use. In these areas, it is imperative to simultaneously consider both aviation safety requirements and the demands of 5G usage. This necessitates the consideration of interference mitigation measures based on power control and angle shutoff techniques. This paper presents an effective and direct analytical framework for interference mitigation measures.

#### 4.6.2. The Integrity and Accuracy of the Model

This paper ensures the integrity and accuracy of the model by referencing extensive work from reputable research institutions. Regarding 5G base station parameters, the paper relies on standards from 3GPP. For interference and antenna modeling, references are made to ITU recommendations. Additionally, interference thresholds are derived from experimental results provided by AVSI and relevant requirements from ICAO. For scenario analysis, this paper draws on guidance provided by RTCA. These institutions provide parameters based on industry standards and experimental findings, ensuring the integrity and accuracy of the model.

#### 4.6.3. The Application of the Deployment Protection Method

**Figure 26:**
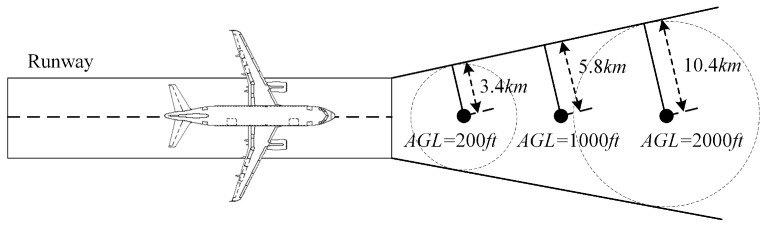
The application of prohibited zones.

The conclusions drawn from this research can be applied to delineate the scope of prohibited zones. Figure 26 (not to scale) provides an illustration of the application of prohibited zones. As the aircraft climbs after takeoff, the radius of the horizontal and vertical prohibited zones gradually increases for altitudes below 2000 ft. Base stations deployed in these zones may interfere with radio altimeters, affecting flight safety. Second, in the prohibited zones, base stations can implement interference mitigation measures, including power control and angle shutoff methods. For example, in Figure 25c, at a range of 5 km at AGL=2000 ft, base stations need only shut off transmission angles of no more than 45° to avoid such interference. This can serve as a technical requirement for base stations in the prohibited zones.

## 5. Conclusions

First, the integrated interference model proposed in the paper is a valuable method for deployment protection. It can be effectively utilized for the analysis of prohibited zones. The method analyzes prohibited zones centered around the aircraft to facilitate the study of the variations of these zones during aircraft movement. By integrating aviation operational safety requirements with standards in telecommunication technology, the method incorporates numerous factors into an integrated model. The analysis culminates in conclusions regarding both horizontal and vertical prohibited zones under typical parameters. Second, this paper analyzes the variations in prohibited zones under various factors, including three types of base station, two types of antenna, two path losses, and a 10° vertical scan angle, combined with changes in aircraft attitude and altitude, as well as ICAO 6 dB margin and the dynamic changes in and interference thresholds, providing a comprehensive understanding of the prohibited zone dynamics. The largest prohibited zone occurs with RMa base stations at AGL=2000 ft and a 30° aircraft pitch\roll angle. This analytical approach enables rapid generation of analysis results for base stations with different technological architectures. Third, this paper also proposes interference mitigation measures within the prohibited zones based on power control and angle shutoff methods. It provides an analytical framework for deploying base stations near runways. This offers a practical basis for policy formulation by mitigating base station interference within the prohibited zones while ensuring aviation safety.

In the future, we can further explore scenarios where multiple base stations near the prohibited zones collectively generate interference. Additionally, we can investigate hybrid approaches combining power control and angle shutoff methods to adapt to emerging technologies such as 5G ATG (Air to Ground) and 5G NTNs (Non-terrestrial Networks).

## Figures and Tables

**Table 2:** Interference thresholds for categories 1, 2, and 3 at 3.93 GHz. *T*: threshold (dBm); *T*_6dB_: threshold with ICAO 6dB margin (dBm).

Altimeter	Frequency (GHz)		Altitude AGL (ft)			
			**200**	**1000**	**2000**	**5000**
Category 1	3.93	*T*	−16	−24	-	−30
		*T*_6dB_	−22	−30	-	−36
Category 2	3.93	*T*	−42	−50	−63	-
		*T*_6dB_	−48	−56	−69	-
Category 3	3.93	*T*	−37	-	-	-
		*T*_6dB_	−43	-	-	-

**Table 3:** BS parameters.

		RMa	UMa	UMi
BS maximum Tx power (dBm)		46	38	38
BS antenna height (m)		35	25	10
Antenna pattern		3GPP TR 38.901		
Omni antenna gain (dBi)		15		
Maximum array gain 16 × 16 (dBi)		29.5		
Channel bandwidth (MHz)		100	100	100
BS maximum PSD (dBm/MHz)		26	18	18
Building height (m)		7	None	None
Street width (m)		25	None	None
LOS shadow fading (dB)		6	4	4
NLOS shadow fading (dB)		8	6	7.82
Maximum vertical scan angle θ (°)		10	10	10

**Table 4:** Radius of Horizontal prohibited zone (*R*: radius without ICAO 6 dB margin (km); *R*_6dB_: radius with ICAO 6 dB margin (km)).

		200 ft		1000 ft		2000 ft	
		R	R **_6dB_**	R	R **_6dB_**	R	R **_6dB_**
Omni Antenna		0.2	0.4	0.9	1.3	2.3	3.2
16 × 16 Beamforming antenna	θ=0°	0.8	1.4	0.4	0.9	2.5	3.4
	θ=10°	0.9	1.5	2.1	3.1	5.0	6.7
	θ=10°, α\β=30°	2.2	3.4	4.2	5.8	8.4	10.4

**Table 5:** Radius of vertical prohibited zone (*R*: radius without ICAO 6 dB margin (km); *R*_6dB_: Radius with ICAO 6 dB margin (km)).

		AGL = 200 ft				AGL = 1000 ft				AGL = 2000 ft			
		R		R **_6dB_**		R		R **_6dB_**		R		R **_6dB_**	
		**0 ft**	**200 ft**	**0 ft**	**200 ft**	**0 ft**	**1000 ft**	**0 ft**	**1000 ft**	**0 ft**	**2000 ft**	**0 ft**	**2000 ft**
Omni Antenna		0.2	0.2	0.4	0.4	0.9	0.5	1.3	1.0	2.3	1.5	3.2	2.6
16 × 16 Beamforming Antenna	θ=0°	0.8	0.8	1.4	1.5	0.4	1.7	0.9	2.8	2.5	4.3	3.4	6.1
	θ=10°	0.9	0.9	1.5	1.5	2.1	1.8	3.1	2.8	5.0	4.5	6.7	6.2
	θ=10°, α\β=30°	2.2	2.2	3.4	3.4	4.2	4.0	5.8	5.6	8.4	8.0	10.4	9.9

## Data Availability

The data presented in this study are available on request from the corresponding author on reasonable request.
